# Using Surveillance of Animal Bite Patients to Decipher Potential Risks of Rabies Exposure From Domestic Animals and Wildlife in Brazil

**DOI:** 10.3389/fpubh.2020.00318

**Published:** 2020-07-22

**Authors:** Julio A. Benavides, Jane Megid, Aline Campos, Katie Hampson

**Affiliations:** ^1^Facultad de Ciencias de la Vida, Universidad Andrés Bello, Santiago, Chile; ^2^Centro de Investigación para la Sustentabilidad, Facultad de Ciencias de la Vida, Universidad Andrés Bello, Santiago, Chile; ^3^Department of Veterinary Hygiene and Public Health, São Paulo State University, Botucatu, Brazil; ^4^Institute of Biodiversity, Animal Health and Comparative Medicine, College of Medical, Veterinary and Life Sciences, University of Glasgow, Glasgow, United Kingdom; ^5^Programa Estadual de Controle e Profilaxia da Raiva, Health Secretary of Rio Grande Do Sul, Porto Alegre, Brazil

**Keywords:** primates, fox, bat, spillover, post-exposure, rabies exposures, public health, zoonoses

## Abstract

Direct contact with domestic animals and wildlife is linked to zoonotic spillover risk. Patients presenting with animal-bite injuries provide a potentially valuable source of surveillance data on rabies viruses that are transmitted primarily by animal bites. Here, we used passive surveillance data of bite patients to identify areas with high potential risk of rabies transmission to humans across Brazil, a highly diverse and populous country, where rabies circulates in a range of species. We analyzed one decade of bite patient data from the national health information system (SINAN) comprising over 500,000 patients attending public health facilities after being bitten by a domestic or wild animal. Our analyses show that, between 2008 and 2016, patients were mostly bitten by domestic dogs (average annual dog bite patients: 502,043 [436,391–544,564], annual incidence per state: 258 dog bites/100,000 persons) and cats (76,512 [56,588–97,580] cat bites, 41 cat bites/100,000/year), but bites from bats (4,172 [3,351–5,365] bat bites, 2.3/100,000/year), primates (3,320 [3,013–3,710] primate bites, 2.0/100,000/year), herbivores (1,908 [1,492–2,298] herbivore bites, 0.9/100,000/year) and foxes (883 [609–1,086] fox bites, 0.6/100,000/year) were also considerable. Incidence of bites due to dogs and herbivores remained relatively stable over the last decade. In contrast bites by cats and bats increased while bites by primates and foxes decreased. Bites by wild animals occurred in all states but were more frequent in the North and Northeast of Brazil, with over 3-fold differences in incidence between states across all animal groups. Most bites reported from domestic animals and wildlife occurred in urban settings (71%), except for bites from foxes, which were higher in rural settings (57%). Based upon the Ministry of Health guidelines, only half of patients received the correct Post-Exposure Prophylaxis following a bite by a suspect rabid animal. We identified areas and species of high-risk for potential zoonotic transmission of rabies in Brazil and reveal that, despite increasing human encroachment into natural ecosystems, only patients reporting bites by bats increased. Our study calls for future research to identity the socio-ecological factors underlying bites and the preventive measures needed to reduce their incidence and potential risk of rabies transmission.

## Introduction

Direct contact with wild and domestic animals is a major driver of zoonotic spillover, defined as the “transmission of a pathogen from a vertebrate animal to a human” (1). Rabies virus (RABV) causes the deadliest known disease and is directly transmitted through the bite of infectious mammals (2, 3). Worldwide, the largest reservoir for rabies and cause of most human rabies deaths is domestic dogs (4). However, following the widespread control of rabies in domestic dog populations, rabies transmitted by wild animals has become the main source of human deaths in the Americas (5, 6). In particular, most human deaths and livestock losses from rabies in the continent are now attributed to spillover from vampire bats, *Desmodus rotundus* (6–8). Foxes (specifically the crab-eating fox *Cerdocyon thous*) and primates (specifically the marmoset *Callithrix jacchus*) are also considered reservoirs of specific RABV variants (9–12), while serological studies show evidence of rabies exposure in several other primate and marsupial species but without evidence of clinical infections (13, 14).

In large and biodiverse countries such as Brazil, rabies circulates among a wide range of wild species including bats, primates, and foxes, complicating the establishment of preventative measures aiming to limit rabies spillover to humans and domestic animals (15–17). The high cost and current uncertainties in the interpretation of serological data among wildlife has restricted the implementation of serology to a small number of wild populations and specific regions of the country (12). In the Northeast region, crab-eating fox and bats have been frequently found exposed or infected, while transmission of RABV from foxes to domestic dogs has been reported (16, 18). Marmosets are also host to a distinct RABV variant in the Northeast region of Brazil (9, 11). In the states of São Paulo and Rio Grande do Sul (RGS) in Southern Brazil, RABV circulation has been detected in vampire bats, insectivorous bats, capuchin monkeys, and crab-eating foxes (12–14, 19–21). These reports suggest the circulation of rabies among different wildlife populations in several regions of the country, posing risks to humans and domestic animals that will depend upon contacts between these populations. Between 2000 and 2017, Brazil reported 188 human rabies cases (22). Among the 46 cases where the rabies variant was identified, 27 cases originated from the common vampire bat variant including three that were transmitted by cats, three cases were from the marmoset variant and 16 cases were from the fox variant (22).

Identifying and reducing direct contact between wild and domestic animals and humans including awareness campaigns to limit contacts with wildlife (e.g., reduce wildlife feeding) may prevent human rabies exposures (23). Patients presenting with animal-bite injuries provide a valuable source of surveillance data on rabies viruses transmitted by bites, including potential exposure risks and evidence of circulation among different reservoir hosts (24). Publicly available data on these patients can allow identification of geographical areas with higher rabies risks that represent a burden to the country's national health system. Brazil's National Health System (SUS) provides universal health coverage to most Brazilians using a network of public hospitals and health facilities. Since 1998 Brazil has used a national health ‘Information System on Diseases of Compulsory Declaration' (SINAN) recording all patients seeking medical care in public health facilities following direct contact with a suspect rabid animal. SINAN records several variables including information on the animal species responsible for the bite, the severity of the bite, whether the biting animal is suspected for rabies, and the Post-Exposure Prophylaxis (PEP) regimen administered to the patient (24, 25).

Using data from SINAN we recently reported that bites from dogs remained relatively stable over the last decade in Brazil and that use of PEP from putatively “rabies-free” states did not decrease despite rabies from domestic dog populations being close to elimination (24). One concern is that unnecessary PEP use may reduce resources available to address risks from the circulation of rabies in alternative wildlife reservoirs, such as bats or primates, which exist in several geographic localities. This is particularly concerning given that vaccine shortages that have been experienced in Brazil during recent years (24). However, bite incidence and the potential risk of rabies from wild animals in Brazil remains poorly understood. The goal of this study was to examine the burden of bites and rabies risk across Brazil posed by both domestic and wild species that are known to transmit rabies. We specifically aimed to (i) compare the incidence of bites from different species across states, (ii) evaluate temporal trends in bites from different species, (iii) assess the extent to which bite incidence is concentrated in urban or rural areas, and (iv) evaluate the appropriateness of PEP use for bites by different species.

## Methods

**Figure 1:**
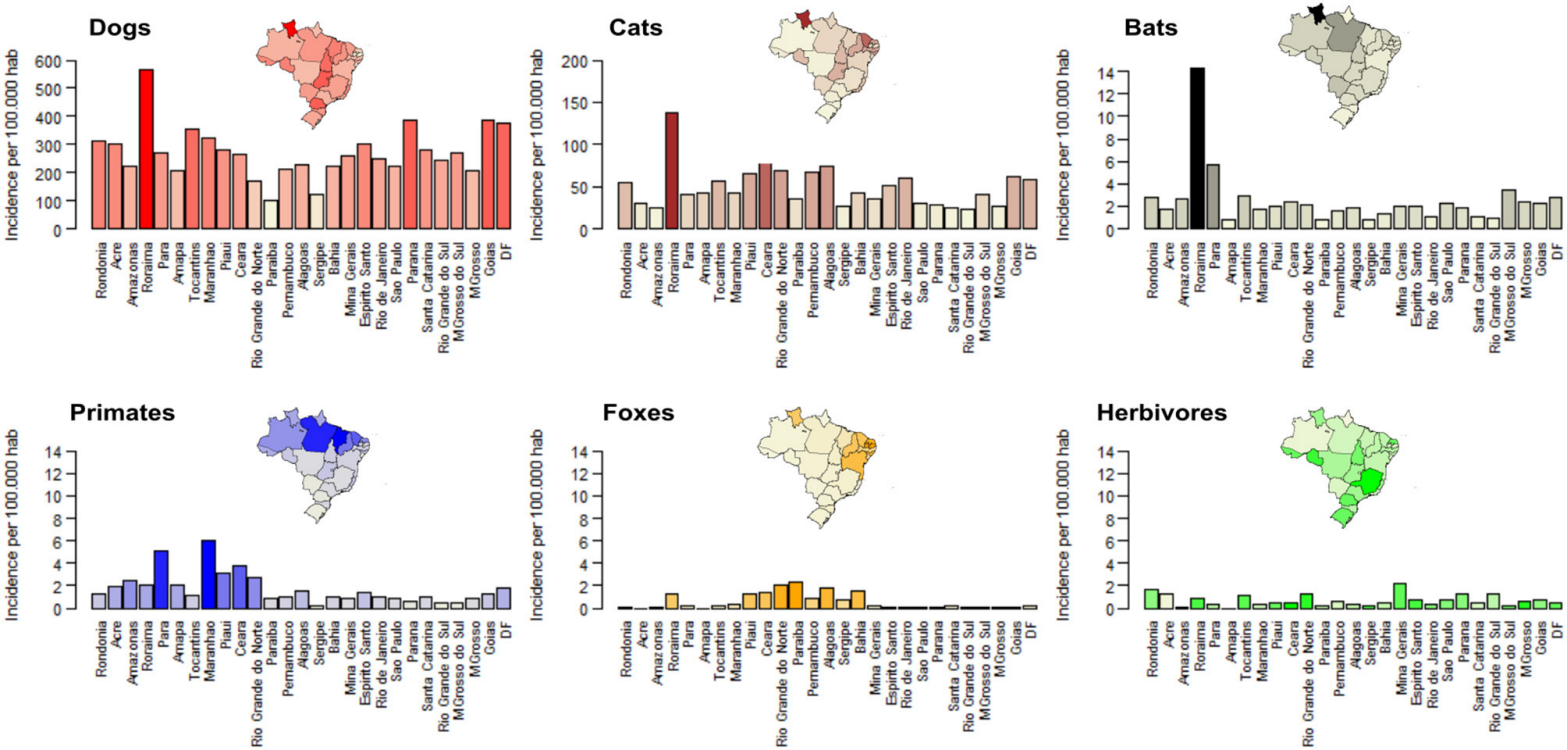
Bite incidence by different species across Brazil in 2016. Each specie's incidence is shown by a different color. The darker the color on each bar, the higher the incidence. Colors of insert maps represent incidence for each state using the same colors as in the bar charts. For comparison, the *y*-axis in the barplots for wildlife ranges from 0 to 14.

Data and spatio-temporal analyses used here are similar to those reported for dog bites in Brazil (24), although data on bites from cats, wildlife, and domestic herbivores have not been previously analyzed. Analyses on dog bites reported in Figures 1, **3**, **5** are presented in our previous work (24) but are compared here to bites from other domestic and wild animals. We obtained data from the “Individual Investigation Reports of Human Anti-rabies Care” form completed by public health workers in Brazil and submitted to SINAN, the national electronic system (26). This form can be completed by any health professional (doctor, nurse, technician) each time a patient seeks care at a public health facility following an animal bite. The form (available here in portuguese: http://portalsinan.saude.gov.br/images/documentos/Agravos/Atendimento%20Anti-rabico/anti_rabico_v5.pdf) includes 60 fields. The SINAN notification (electronic data) of the aggression (bite) must be sent weekly from municipality to state level, and every 2 weeks from state to federal level. All cases must be concluded within 60 days. The patient assessment includes identifying the species responsible for the bite, and the use and follow-up of PEP after assessment by a doctor or nurse.

Forms are divided into five sections including (i) general data on the health unit, (ii) patient characteristics, (iii) location of patient's residency and health care unit (e.g., municipality and state), (iv) epidemiological information including the animal responsible for the bite and the prior PEP history of the patient as well as (v) the PEP recommended and administered to the patient and the status of the animal after a potential 10-day observation period. Data were obtained for Brazil from 2008 to 2016 via an online request to the Ministry of Health through the Electronic System of Citizen's information (e-SIC, https://esic.cgu.gov.br/sistema/site/index.aspx, e-SIC request number: 2564134). Empty fields were assumed to have not been completed and were shown in the analysis as “no data available.”

Data was analyzed using R 3.6.1 (27).

### Temporal and Spatial Trends in Bite Incidence by Species

We first explored whether bite incidence, i.e., the number of patients seeking health care after a bite per 100,000 habitants, varied across states and between 2008 and 2016 according to the species of the biting animal. We calculated bite incidence as the number of completed reports divided by the total human population of each state, extracted from publicly available census data from the Brazilian Institute of Geography and Statistics (IBGE) (https://www.ibge.gov.br/). To determine whether different species pose a similar health burden across states we also tested for correlations in incidence. We used a non-parametric Spearman's correlation test with the *cor.test* function in R, since data was not normally distributed across states. Using the field indicating whether a patient was bitten in an urban or rural district, we further compared the incidence of bites for each species between urban and rural districts.

### Administration of PEP According to Ministry of Health Guidelines

To evaluate if PEP is being effectively delivered in Brazil, we compared the PEP given to each patient recorded in SINAN in 2016 with the appropriate PEP for the same patient according to the Brazilian's Ministry of Health (MoH) prophylaxis guidelines from 2014, based upon the variables reported in SINAN. The SINAN form includes seven PEP recommendations: “Pre-exposure prophylaxis,” “No prophylaxis,” “Observe the animal (if dog or cat) for 10 days but no vaccine or serum (immunoglobulin)”, “Observe the animal and administer vaccine,” “Administer vaccine but no serum,” “Administer vaccine and serum,” “Re-exposure prophylaxis.” Based on PEP guidelines, wild animals were considered as rabid animals and no observation period was requested. We used an algorithm to calculate “appropriate” PEP considering the risk assessment data from each SINAN form following the MoH guidelines. The MoH guidelines are based on three criteria: (i) bite/incident severity, (ii) animal species and dog/cat status before and after the 10-day period observation, and (iii) previous PEP history (vaccination/vaccine titers). Details of these guidelines are available in the Table S1. First, the algorithm differentiates each SINAN form as either a “severe” or “mild” incident according to three variables specified in the MoH guidance: the type of exposure, the position of the exposure, and the injury and type of injury (Table S1). If an exposure did not fulfill the criteria to be classified as a “severe incident,” it was classified as a “mild” incident. We considered incidents to be “mild” if the form details about the exposure were completed as “other or ignored,” if the “position of exposure” was “unknown,” or if the “injury” or the “type of injury” was “ignored.” Second, the algorithm further discriminated PEP based on whether the animal was considered “healthy,” “rabies suspicious,” “rabid,” or “dead/disappeared.” Finally, the algorithm separated patients on whether they had received complete PEP previously, which reduced their subsequent PEP requirements.

## Results

### Spatiotemporal Trends

Between 2008 and 2016, 82.3% of all bite patients in Brazil were attributed to bites from dogs, 12.5% from cats, 1.4% from wild animals, 0.3% from herbivores, 2.7% from other unidentified animals, whilst 0.8% of records did not have information recorded on the species of biting animal. Among wild animals, 49.8% of bites were attributed to bats, 39.6% to primates and 10.5% to foxes. The average number of patients bitten by dogs over this period was: 502,043 [95% CI: 436,391–544,564], with an annual incidence per state of 258 dog bites/100,000 people. The number of people bitten by other species were generally much lower, but on average there were 76,512 cat bites per year [95% CI: 56,588–97,580], incidence: 41 cat bites/100,000 persons, followed by 4,172 bat bites [95% CI: 3,351–5,365] or 2.3 bat bites/100,000 persons, 3,320 primate bites [95% CI: 3,013–3,710] or 2.0 primate bites/100,000 persons, 1,908 herbivore bites [1,492–2,298], or 0.9 herbivore bites/100,000 persons and 883 fox bites [95% CI: 609–1,086] or 0.6 fox bites/100,000 persons (Figure 1).

**Figure 2:**
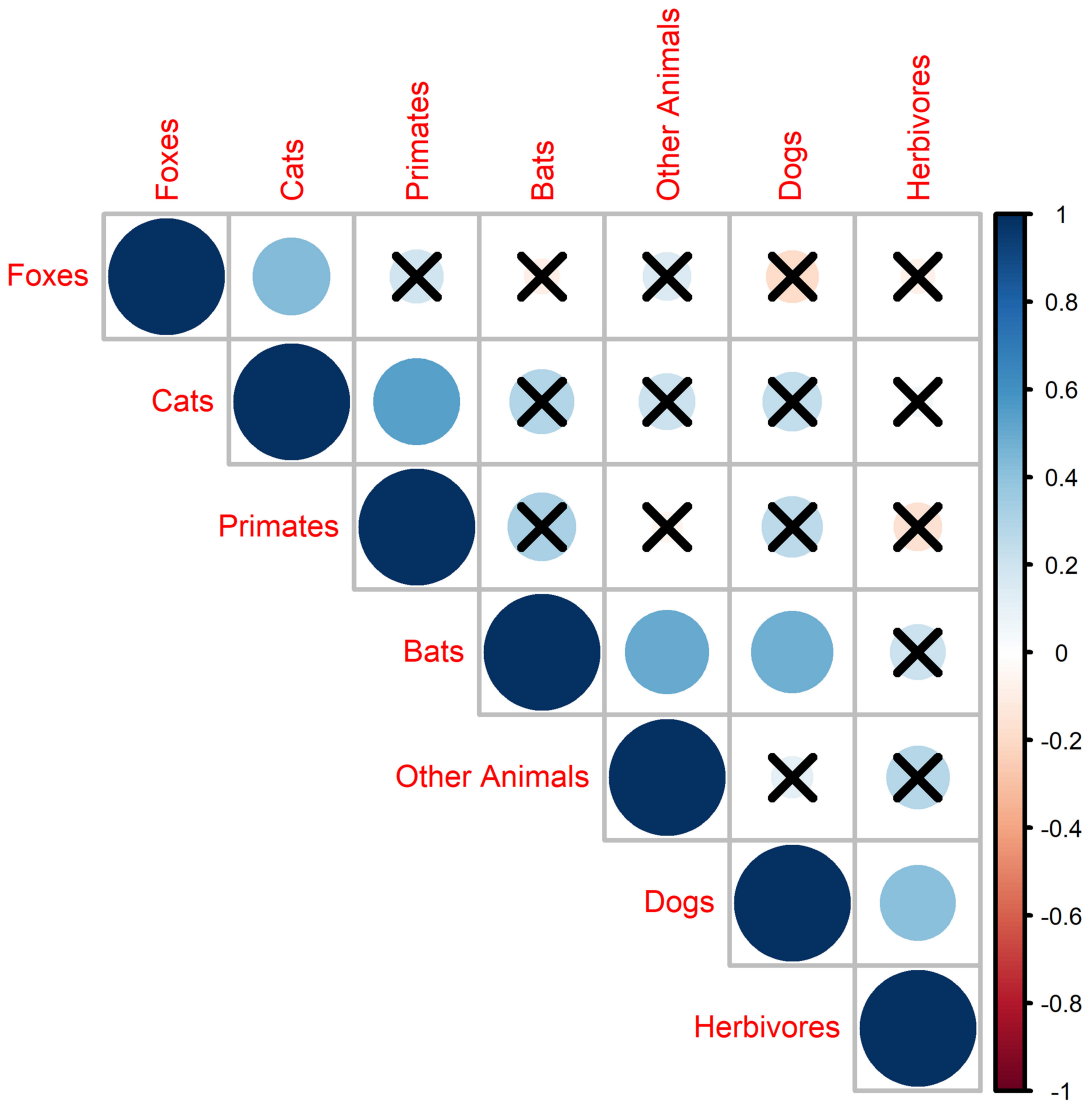
Correlation in bite incidence across states. Circle size is proportional to the value of the Spearman's correlation rho. Blue colors indicate a positive correlation and red colors a negative correlation. Crosses over circles indicate that the relationship was not statistically significant.

Bites from domestic and wild animals occurred all over Brazil but bite incidence varied considerably between states, with more than three-fold differences in incidence between states across all species (Figure 1). Overall, the northern region of Brazil had a higher burden of bites (Figure 1). For example, Roraima had the highest bite incidence for dogs, cats, and bats, while primate bites were reported mostly in the North (states of Maranhão and Pará) and bites from foxes were predominantly reported from the North East region (states of Paraiba and Rio Grande do Norte; Figure 1). Bites of several species were significantly correlated across states including bites from cats and primates (Spearman test, rho = 0.54, *p* < 0.01), cats and foxes (Spearman test, rho = 0.43, *p* < 0.02), and bats with dogs (Spearman test, rho = 0.49, *p* = 0.01) and other animals (Spearman test, rho = 0.43, *p* = 0.02; Figure 2).

**Figure 3:**
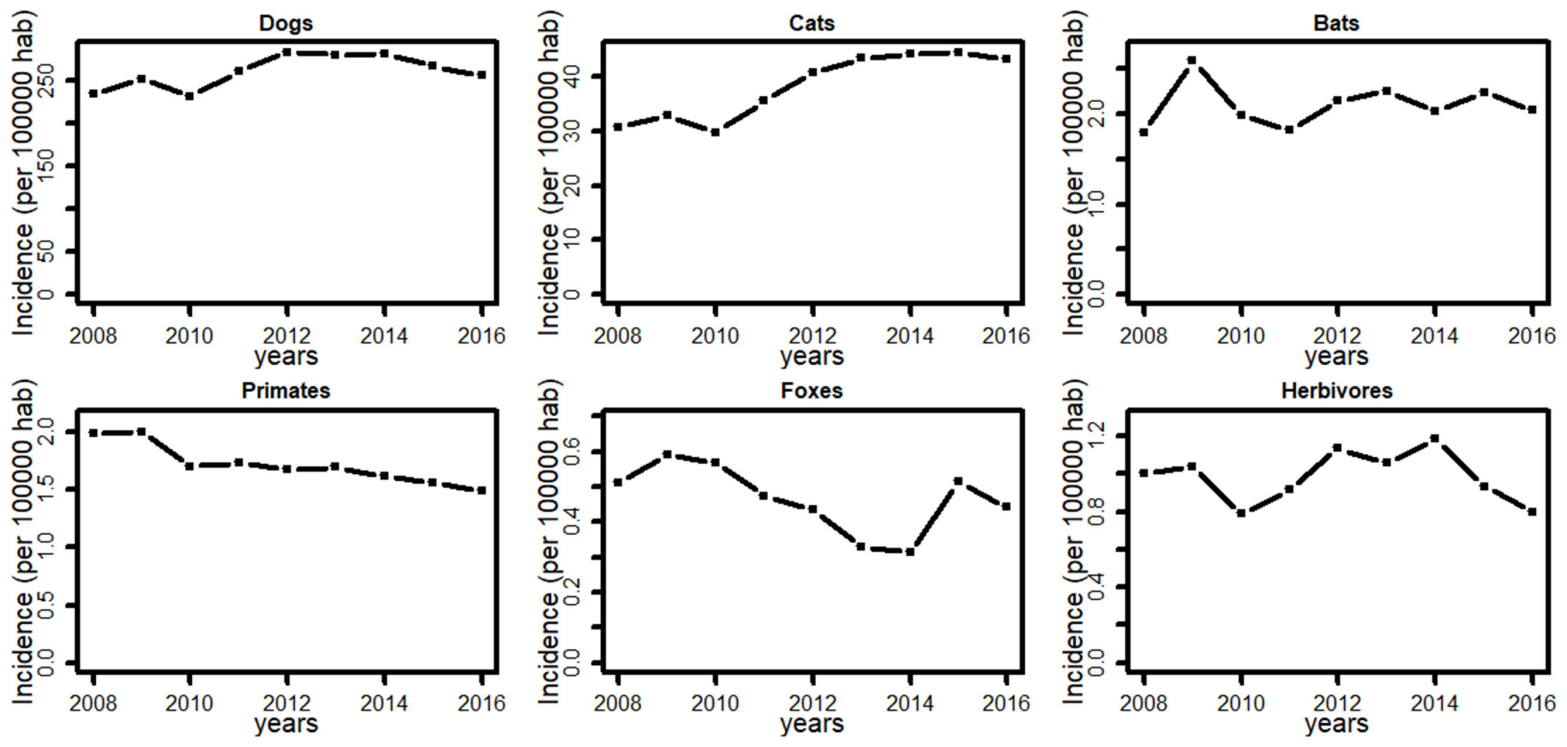
Average Bite incidence in Brazil between 2008 and 2016 for different species.

Incidence of bites due to dogs and herbivores remained relatively stable over the last decade. In contrast, bites from cats increased by 56%, and bites from bats increased by 13% between 2008 and 2016, while bites from primates decreased by 34% and bites from foxes decreased by 16% (Figure 3). Likewise, the number of municipalities reporting bites increased by 11% (3,742–4,169) for cats, by 13% (1,044–1,184) for bats and decreased by 14% (1,097–947) for primates, and 7% (455–422) for foxes.

### Rural vs. Urban

**Figure 4:**
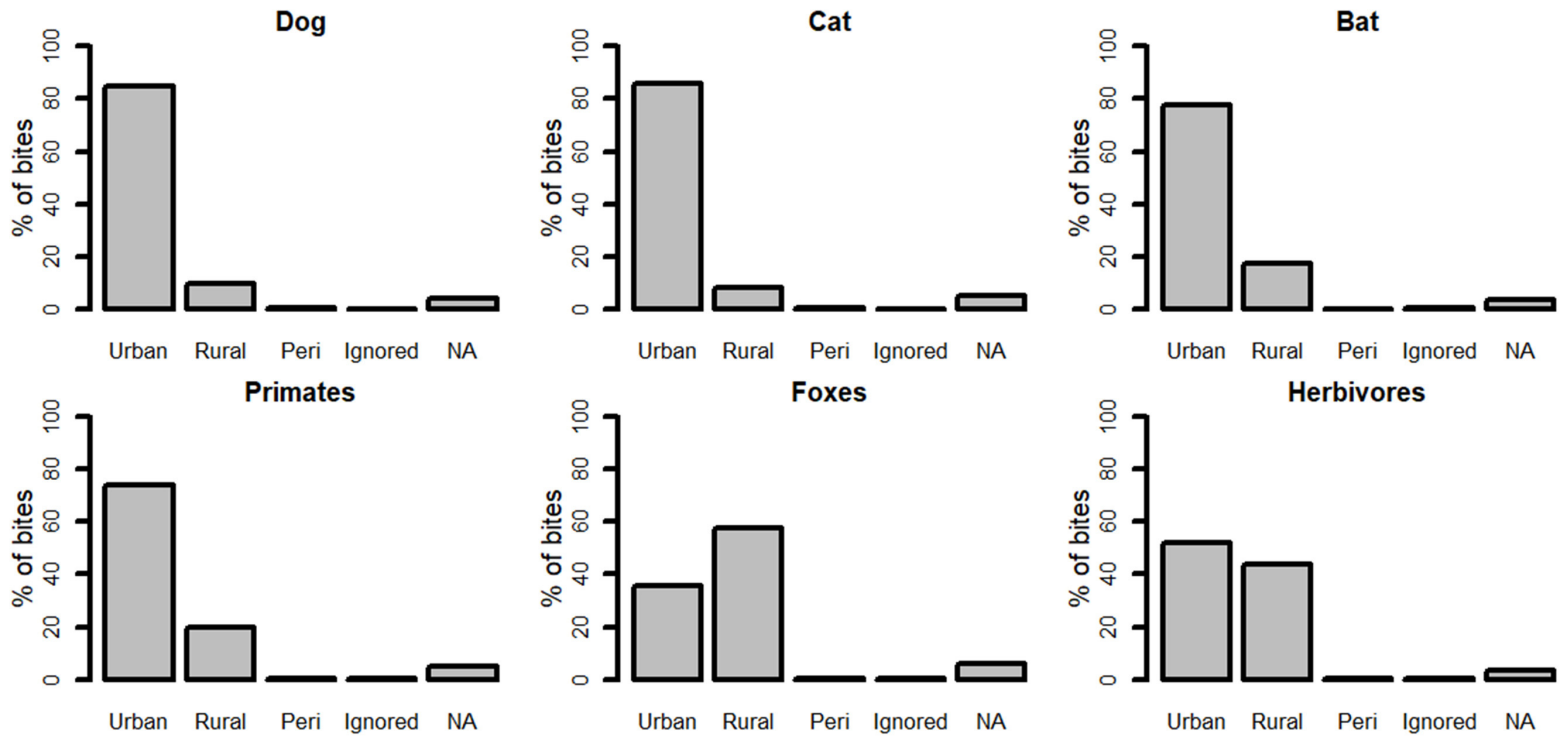
Percentage of patients reporting bites from urban, rural, and peri-urban areas in 2016 according to the species of biting animal.

Overall, 84.3% of bites were reported in urban areas, with just 10.0% from rural areas, 0.7% from peri-urban areas, and 5% of forms did not have this information completed. More than 85% of bites from dogs and cats were reported from urban areas (Figure 4). Most bites from bats (78%), from primates (74%) and from herbivores (52%) were reported from urban areas (Figure 4). In contrast, just 36% of bites from foxes occurred in urban areas (Figure 4).

### Appropriate PEP Administration According to the Brazilian MoH

**Figure 5:**
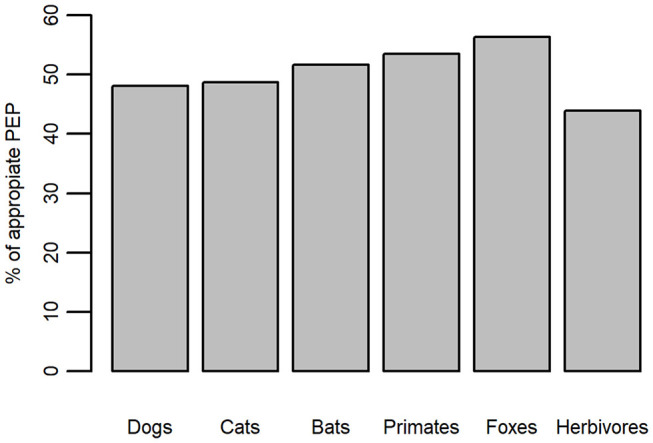
Percentage of appropriately administered PEP for bites by different species in 2016 according to the Ministry of Health guidelines.

For bites involving wild animals and herbivores, the MoH recommends that patients involved in a “mild incident” receive PEP including vaccination while patients involved in a “severe incident” require both vaccination and serum. Therefore, any wild animal that can transmit rabies is considered the equivalent of a rabid dog or cat. By applying the algorithm described above to bite data from 2016, our analyses showed that patients received appropriate PEP in 50% of bites irrespective of the species of biting animal. Appropriate PEP was given to 48.1% of bites involving dogs, 48.7% of cat bites, 51.6% of bat bites, 53.5% of primate bites, 56.3% of fox bites, and 44% of herbivore bites (Figure 5).

## Discussion

Rabies in wildlife is an emerging challenge in Latin America (28). However, surveillance in wild animal populations is challenging and hotspots of potential spillover risk remain poorly understood for most wild reservoirs of rabies viruses. Our previous work described high incidence of dog bites in Brazil that have remained stable in the past decade but uneven across states, with around half of PEP for dog bites not administered appropriately (24). This study shows that although bite incidence due to wild animals is much lower than for dogs and cats, there are still substantial numbers of patients seeking health care due to wild animal bites all over Brazil. Overall bite incidence due to primates and bats was similar in magnitude, but three times higher than due to foxes. Bites from wildlife were geographically localized, with bites from primates concentrated in the north of Brazil, bites from foxes in the Northeast, and highest incidence of bat bites in Roraima state. Bite incidence between species was correlated at the region level between cats and primates or foxes, and between dogs and bats. Despite increased human encroachment into natural areas, our results show that only bites from bats increased over the last decade (by 13%) while bites from both primates and foxes decreased. Most bites from domestic and wild animals were reported in urban areas, except for bites by foxes. Similar to our previous findings in dogs (24), appropriate PEP was given to only about half of patients attending health care after being bitten by cats and wild animals, highlighting the need to improve health worker‘s knowledge on PEP following a bite from domestic and wild animals.

Estimates of direct contact between humans and wild animals are rare worldwide, limiting our ability to predict the rate and location of emerging zoonotic diseases (1, 29). Our results take advantage of the publicly available SINAN database to estimate and compare bite incidence across states of Brazil, which is the route of human exposure to rabies. As expected, bite incidence in wild animals (0.6–2.3 bites/100,000 hab) was much lower compared to bites from dogs (258 bites/100,000) reported in our previous study (24) or cats (51 bites/100,000). Yet, despite the relatively low incidence, bites from primates and bats affected patients in all states. The low incidence of bites by wildlife in some regions should be interpreted with caution, given that levels of under-reporting are unknown and could be particularly high for isolated populations around natural areas such as indigenous communities, frequently affected by bites from vampire bats (30). The geographical differences in bite incidence imposes an uneven burden to the public health system across the country. Differences between states could reflect the distribution of wildlife populations (e.g., higher abundance of primates and foxes in the North), as well as socio-cultural differences that affects human-wildlife interactions including differences in animal feeding (e.g., feeding in houses for the pet trade or at recreation sites for marmosets or capuchin monkeys) or habitat suitability for opportunistic species such as bats and foxes in urban areas. Similarly, correlations in bite incidence across states for different species (e.g., cats and primates) could be explained by the abundance of those species, similarities in health seeking behavior or reflect spillover risks of rabies between species that might warrant further investigation.

Increased contact between people and wildlife due to human activities such as hunting, agriculture, deforestation, and urbanization is a worldwide problem resulting in the emergence of diseases critical for public health including HIV-SIV, malaria, Ebola, and influenza (31–33). Thus, we could expect that in Brazil, one of the most biodiverse countries worldwide with an increase urbanization, agriculture, and deforestation, close contact between humans and wild animals would also increase over the last decade (34, 35). Our results show that, at least for patients attending health care facilities, bite incidence from primates (-34%) and foxes (-16%) has decreased since 2008, while only bites from bats (+13%) and cats (+56%) increased. The number of municipalities reporting bites followed the same pattern, suggesting that these temporal changes reflect corresponding increases or reductions in the spatial extent of direct contact between humans and wild animals. Overall, our study calls for a better understanding of the drivers behind these temporal trends, specifically increasing bites from bats and cats, and reductions in bites from primates and foxes.

Despite increased urbanization, reductions in bites from wild primates and foxes could reflect the effectiveness of regional educational campaigns aiming to reduce these high-risk contacts. In contrast, the observed increased in bites from bats could reflect range expansions for species such as vampire bats, due to climatic change (7) and increased availability of (man-made) roost sites with urbanization (36, 37). This could also help to explain why bites from bats, primates, and even herbivores are more frequently reported in patients living in urban areas. However, predominance of bites in urban areas (also observed for domestic animals) could also reflect under-reporting of bites in rural areas due to both fewer health centers and lower perceived risks.

Our study shows that 1.4% of bites reported in SINAN are attributed to wild animals compared to 94% of bites attributed to companion animals (dogs and cats). Though bites by wild animals present a relatively low burden for health care systems, they likely represent a higher risk of rabies to humans given continued circulation of several rabies virus variants in these species, the absence of successful strategies to control vampire bat rabies (8), or rabies in primates and foxes. There may be scope for oral rabies vaccines to be used to control rabies, particularly in foxes in Latin America if strategies can be adapted from those that have proven successful in Europe (38). Alarmingly, we estimate that only half of bites involving wild animals received appropriate PEP according to MoH Guideline's during that period. Like domestic animals, this could reflect poor knowledge of health care personnel on appropriate PEP administration but also lack of PEP availability in remote areas where wildlife species are abundant. In the case of bites by herbivores, which have the lowest percentage of correct PEP administration (44%), this may be due to poor knowledge of the risk of rabies transmission from herbivores and the epidemiological situation whereby herbivores are frequently infected by vampire bat rabies. Wild species can also transmit several other viral diseases to humans, with bats and primates considered reservoirs for many zoonotic emerging diseases (39, 40). Given the current circulation of rabies in different wildlife species as well as ongoing vaccine and immunoglobulin shortages (41), there is an urgent need to improve PEP administration for bites by wild species to avoid rabies fatalities.

Given the difficulty to control rabies among wildlife reservoirs, prevention measures aiming to reduce human rabies exposures could focus on reducing bites. Following our previous work on dogs (24), this study provides a first estimate of bites from cats and wildlife species in Brazil, showing the uneven incidence across the country and the relatively low level of adequate PEP administration. Despite thousands of bites per year from domestic and wild animals, <10 human rabies cases were reported annually to SINAN during the same period (22). The public health risk of rabies could therefore be limited by low circulation of rabies in these animal reservoirs. Alternatively, rabies circulation in some species (e.g., bats) could be high but PEP administration, although inadequate, is still preventing the development of rabies in patients that were bitten by rabid animals. Further reducing exposure risk will require different strategies adapted at the region level aiming to reduce bites and will benefit from the implementation of a “One health” approach. Community-based surveys could also help to identify the socio-ecological factors underlying bites and under-reporting. Rabies surveillance in wild reservoir populations could be guided by the incidence data that we report which highlight areas with the highest bite rates, whilst also carefully considering potential under-reporting in remote communities. Improvements of the SINAN system could include further detailing the wild species responsible for the bite as well as the reason for the incident. This could in turn inform educational campaigns aiming to reduce contact with wild animals such as primates due to the pet trade, feeding of primates and foxes and reducing roost sites for bats in urban areas.

## Data Availability Statement

The datasets generated for this study will not be made publicly available. Data were obtained for Brazil from 2008 to 2016 by requesting the data online to the Ministry of Health through the Electronic System of Citizen's information (e-SIC, https://esic.cgu.gov.br/sistema/site/index.aspx, e-SIC request number: 2564134). Data was requested from the Ministry of Health but cannot be shared publicly.

## Ethics Statement

This project was approved by the Brazilian Ministry of Health's Ethics Committee (Plataforma Brazil, CAE # 94081818.0.0000.5411) through the Ethics Committee of the Faculty of Medicine of the São Paulo State University (UNESP)—Botucatu.

## Author Contributions

JB conceived, designed the study, and analyzed the data. JB and KH drafted the paper. JB, KH, AC, and JM edited the paper. All authors read, commented, and approved the final manuscript. All authors contributed to the article and approved the submitted version.

## Conflict of Interest

The authors declare that the research was conducted in the absence of any commercial or financial relationships that could be construed as a potential conflict of interest.

## Supplementary Material

Table S1 Brazilian Ministry of Health guidelines for rabies prophylaxis.
